# Determinants of implementing the 15-method in Danish general practice using the consolidated framework for implementation science

**DOI:** 10.1186/s13722-025-00571-0

**Published:** 2025-05-16

**Authors:** Peter Næsborg Schøler, Jens Søndergaard, Sanne Rasmussen, Kristina Hasselbalch Volke, Per Nilsen, Anette Søgaard Nielsen

**Affiliations:** 1 Unit for Clinical Alcohol Research, Research Unit of Psychiatry, Department of Clinical Research, University of Southern Denmark, Odense, Denmark; 2 Research Unit of General Practice Odense and Esbjerg, Department of Public Health, University of Southern Denmark, Odense, Denmark; 3 Department of Psychiatry Odense, Mental Health Services Region of Southern Denmark, Odense, Denmark; 4 Department of Health, Medicine and Caring Sciences, Linköping University, Linköping, Sweden; 5 (BRIDGE), Brain Research - Inter Disciplinary Guided Excellence, University of Southern Denmark, Odense, Denmark

**Keywords:** Screening and brief intervention, Consolidated framework for implementation research, Alcohol intervention, Implementation science, Primary care

## Abstract

**Background:**

Excessive alcohol consumption is a significant global health issue, often unaddressed in primary care. The 15-method, a three-step opportunistic screening and treatment tool premised on Motivational Interviewing and integrated within the Screening, Brief Intervention and Referral to Treatment framework, offers a structured approach for healthcare professionals to identify and treat alcohol-related problems. The present study aimed to assess healthcare professionals’ perceptions of determinants for early-stage implementation of the 15-method in Danish general practice and to classify these determinants using the Consolidated Framework for Implementation Research (CFIR).

**Methods:**

This qualitative study involved individual interviews and group interviews with general practitioners and nurses (*N* = 28) from 12 general practices participating in the Identification and Treatment of Alcohol Problems in Primary Care (iTAPP) study, a stepped-wedge cluster randomized controlled trial evaluating the effectiveness of the 15-method in Danish general practice. Interviews were semi-structured, guided by the CFIR framework, and analyzed using directed content analysis. Determinants were rated for their influence on implementation.

**Results:**

Key facilitators included the 15-method’s adaptability, strong evidence base, relative advantage, and compatibility with existing practices. Barriers included structural characteristics in the practices and local conditions. A central finding revealed a tension between patients’ motivation and healthcare professionals’ opportunities and capabilities to deliver the 15-method. Mixed determinants highlighted the complexity of implementing the 15-method across diverse practices.

**Conclusion:**

Implementing the 15-method in Danish general practice is feasible but requires addressing specific barriers and leveraging facilitators. A multifaceted implementation strategy tailored to individual practices may be necessary to address the variations in contexts and resources across different practices with an emphasis on increasing healthcare professionals’ capabilities and opportunities to deliver the intervention.

**Supplementary Information:**

The online version contains supplementary material available at 10.1186/s13722-025-00571-0.

## Introduction

Excessive alcohol consumption is globally a leading cause mortality and morbidity [1]. Mild to moderate alcohol problems often go unaddressed, as individuals with less severe problems rarely seek treatment [2] and less obvious symptoms tend to be overlooked [3]. As most alcohol problems are mild to moderate, addressing these issues earlier could yield substantial public health benefits [4, 5].

Screening and brief intervention (SBI) for alcohol problems has proven effective in primary care and holds promise due to frequent patient interactions [4, 6, 7]. General practice is particularly well-positioned for implementing the Screening, Brief Intervention, and Referral-to-Treatment (SBIRT) approach, given its continuity of care, coordinated treatment, and generalist approach [3, 4, 8–11]. However, research indicates that the Referral-to-Treatment component has limited efficacy in increasing patient engagement with specialized alcohol-treatment services after the initial intervention [12]. Moreover, implementing sustainable methods to address and treat alcohol problems in general practice is challenging as several factors hinder implementation and sustainability [11, 13, 14], including patients’ fear of stigmatization [15], time constraints and lack of training among healthcare professionals (HCPs) [11], insufficient leadership support, and an organizational culture that does not facilitate work with alcohol issues [16–18].

The 15-method [19] offers an alternative to the traditional SBIRT approach, by integrating screening, intervention, and treatment within the same primary care setting. This method offers HCPs in general practice a manual-based tool to identify and treat alcohol-related problems using evidence-based techniques from Motivational Interviewing and Cognitive Behavioral Therapy. The method is premised on opportunistic screening, where HCPs screen the patient for alcohol-related problems if the patient presents with symptoms that might be related to alcohol. The HCP can utilize the Alcohol Use Disorder Identification Test (AUDIT) [20] as screening tool. As the second step, the HCP engage the patient in a structured consultation, similar to the Drinkers Check-Up [21], to explore patient symptoms in relation to alcohol use. If needed, the HCP offers the patient treatment through one to four structured consultations, including patient homework assignments and pharmacological treatment when appropriate. Treatment with the 15-method is targeted patients with an AUDIT score of 15 points or more, hence the name. Notably, all steps, including treatment, are delivered in the same primary care setting. The method’s effectiveness in Swedish general practice has been found to be non-inferior to specialist treatment [22, 23].

After feasibility testing and adjustment to Danish primary care [24, 25], the effectiveness of the 15-method in Danish general practice is now being tested in The Identification and Treatment of Alcohol Problems in Primary Care (iTAPP) Study [26], a randomized controlled trial involving 21 Danish general practices. The iTAPP study is based on the Medical Research Council (MRC) guidance on complex interventions [27], involving an implementation evaluation aimed to identify factors that might influence the implementation of the method and impact study outcomes [28]. The present study aimed to assess healthcare professionals’ perceptions of determinants for early-stage implementation of the 15-method in Danish general practice and to classify these determinants using the Consolidated Framework for Implementation Research (CFIR) [29].

## Methods and material

### Design

The present study was a qualitative interview study with general practitioners (GPs) and nurses in general practice in Denmark conducted as part of the iTAPP study as an evaluation of determinants influencing the implementation of the 15-method. We used the Consolidated Framework for Implementation Research (CFIR) [29] to guide data collection and analyses of determinants. The CFIR framework synthesizes multiple implementation theories and frameworks into five determinant domains consisting of barriers and facilitators that have been found to influence implementation of various practices [25].

The iTAPP study was designed as a stepped-wedge cluster randomized controlled trial in which four groups, i.e. clusters, of practices were randomized to four different launch times for implementation of the 15-method. All practices began with a three-month baseline period prior to training in the 15-method and were set to launch in three-to-four-month increments. Training in the 15-method lasted three hours and included suggestions for implementation strategy, measures, and goals. HCPs were encouraged to start using the 15-method immediately after training, meaning that there was no designated ‘implementation period’ before adding the 15-method into their routine practice. A detailed description of the iTAPP study and 15-method training has been described elsewhere [26].

### Setting

The present study was conducted in 12 of the 21 general practices participating in the iTAPP study. The general practices are located in the Region of Southern Denmark which encompasses 1,2 million inhabitants [30], 345 general practices [31] with an average of 2, 3 GPs working in each practice. The average GP in this region has 1.541 affiliated patients, and 99% of Danish residents are listed with a general practice [30, 32]. Treatment and consultations are free of charge for the patient. The GP serves as first-line provider and gatekeeper to the secondary health care system [33]. Practices in the iTAPP study were located in both urban and rural areas and included solo practices (one GP) as well as partnership practices (more GPs with shared ownership) [26, 32]. GPs often hold both the managerial and ownership positions in their respective practices but may have additional GPs without ownership employed as part of their staff.

### Recruitment

We recruited HCPs through their participation in the 15-method training session conducted as part of the iTAPP study. The interviews for the present study were outlined in the agreement signed between practice heads and The University of Southern Denmark for the iTAPP study. We recruited HCPs from all practices in cluster one and cluster two in the iTAPP study. We further aimed to include a minimum of one GP in a managerial position and one nurse from each practice to provide insights from both a managerial and a staff perspective.

### The 15-method

The 15-method consists of three steps to address alcohol-related problems. The first step is opportunistic screening and brief advice. The HCP screens for alcohol-related issues during regular consultations if the patient presents physical or mental symptoms that may be affected or caused by alcohol [34]. Screening tools include the AUDIT and biological markers like Alanin-Amino Tranferase (ALAT) and Gamma-Glutamyl Transferase (GGT). If alcohol is found to be relevant for the patient’s situation, the HCP can recommend a follow-up. The patient may also fill in the AUDIT questionnaire between consultations as a link between screening and follow-up.

The second step involves assessment and feedback based on AUDIT scores and biological markers. The HCP provides feedback on the patient’s alcohol consumption and its potential consequences in relation to relevant symptoms. This step aims to enhance the patient’s motivation to change alcohol habits through reflection and feedback. Additional questionnaires such as one-week Timeline Follow-Back [35], The Short Alcohol Dependence Data Questionnaire [36], and the International Classification of Diseases 10th revision (ICD-10) criteria for alcohol dependence [37] may be used to further assess alcohol habits and the HCP can screen for the use of other addictive substances (nicotine, narcotics, benzodiazepines, and opioids).

The third step is comprised of treatment based on Motivational Interviewing [38], Cognitive Behavioral Therapy, and Guided Self-Change [39, 40]. This involves up to three treatment sessions and a follow-up evaluation, using tools like an alcohol diary and homework assignments between sessions for reflection, goal setting, self-monitoring, and identification of risk situations. The HCP and patient determine the treatment intensity and goals through shared decision-making [41], with concomitant pharmacological treatment available following national guidelines (Disulfiram, Acamprosate, Nalmefene, or Naltrexone).

The 15-method material includes HCP manual, quick guide, and homepage. Further, a patient logbook, homework assignments, AUDIT questionnaires, and materials to encourage discussions about alcohol habits (icebreakers) including posters, bottles, and flyers. Supplementary File 1 features examples of the icebreakers. Training of HCPs is conducted through academic detailing [42, 43], focusing on recognizing alcohol-related symptoms, using the 15-method materials, and applying Motivational Interviewing techniques. The 15-method, and its use in the iTAPP study, has also been described in detail elsewhere [19, 26, 44].

### Implementation determinant framework

We selected the CFIR for three main reasons. First, its flexibility allows for application across diverse settings. Second, its multilevel approach to assessing implementation determinants aligns well with the nature of an SBI intervention, which represents a discrete clinical practice change affecting multiple levels, from individual behavior to organizational processes. Third, we prioritized the evaluation of contextual factors, given the variability inherent across multiple practice settings. The CFIR has been comprehensively detailed in previous literature [29, 45]. In essence, the CFIR synthesizes key determinants from various implementation theories at a “meta-theoretical” level but does not delineate the interrelationship among these theories or propose specific hypotheses regarding the potential interactions between determinants [46]. The framework consists of five domains encompassing 48 constructs and 19 sub-constructs, i.e. determinants, known to influence implementation effectiveness [29]. Domain I, *Innovation*, focuses on determinants influential to any “idea, practice, or objective perceived as new” following Rogers’ definition [47], e.g. the perceived complexity of the innovation. Domain II, *Outer Setting*, can include multiple levels and represents the setting in which the inner setting is located, e.g. a state, system or region. Domain III, *Inner Setting*, focuses on the implementation setting within the outer setting and may also include multiple levels or units, e.g. classrooms, practices, or teams. Domain IV, *Individuals*, includes a subdomain focusing on the roles of the individuals, and a subdomain focusing on the characteristics of the individual, e.g. needs, capabilities, and opportunities. Finally, Domain V, *Implementation Process*, assesses the implementation activities and strategies used, e.g. the level of planning or tailoring strategies.

In the present study, the *Innovation* refers to the 15-method, the *Outer Setting* refers to the Region of Southern Denmark and the general healthcare system in Denmark, and the *Inner Setting* refers to the specific practices participating in the iTAPP study.

Notably, implementation determinants refer to factors that may influence implementation success such as provider attitudes, patient motivation, and organizational support, whereas implementation outcome measures refer to measurable indicators of implementation success such as fidelity scores, adoption rates, and penetration measures [48, 49] which were not assessed in the present study.

### Data collection

We collected data in two rounds of interviews between May and December 2023. The first round of interviews included HCPs from all practices in cluster one, at which point they had been using the 15-method for approximately 6–8 weeks. The second round included HCPs from all practices in cluster two, along with follow-up interviews with most HCPs from cluster one to be able to identify potential changes in determinants over time. By the time of the second interview round, HCPs in cluster two had used the 15-method for 11–13 weeks, while those in cluster one had been using it for 32–34 weeks. One practice in cluster one could not participate in the follow-up due to withdrawal from the iTAPP study (low staff resources and lack of time). Additionally, one GP was unavailable during follow-up so a nurse from the same practice participated instead (Table 1).

**Table 1:** Overview of interview participants

Practice number	Cluster number in the iTAPP study	Number of interviews (*N*=17)	Number of participating HCP in interview round one (*n*=12)	Number of participating HCP in interview round two (*n*=25)	Number of HCP in the practice participating in the iTAPP study / total number of HCP in the practice ^a^	Type of interview
1	1^b^	2	1 GP	1 nurse	2 / 2	Individual. Video.
2	2^b^	1	-	4 GPs	6 / 9	Focus group. Video.
3	1	2	1 GP	1 GP (re-interview)	1 / 2	Individual. First in person, second via video.
4	1	1	1 nurse	(discontinued)	1 / 5	Individual. Video.
5	1	2	2 nurses	2 nurses (re-interview), 1 GP	3 / 6	Focus group. Video.
6	1	2	1 GP, 2 nurses	2 nurses (re-interview)	5 / 6	Focus group. First in person, second via video.
7	1	2	2 GPs, 2 nurses	2 GPs (re-interview), 2 nurses (re-interview)	4 / 4	Focus group. In person.
8	2	1	-	1 GP, 2 nurses	3 / 4	Focus group. In person.
9	2	1	-	1 GP, 1 nurse	8 / 8	Focus group. In person.
10	2	1	-	1 GP	7 /8	Individual. In person.
11	2	1	-	2 GPs	3 / 3	Focus group. Video.
12	2	1	-	2 GPs	4 / 4	Focus group. Video.

Notes: GP, General practitioner. HCP, Healthcare professional. ^a^ HCP refers to general practitioners, nurses, and social workers participating in the iTAPP study, excluding secretaries, short-term interns, student workers, and laboratory assistants. ^b^ The practice participated in the feasibility study of the 15-method in Danish general practice leading up to the iTAPP study and continued as participating practice in the iTAPP study

We conducted both group interviews and individual interviews, both in-person and via video. We used this varied approach to accommodate HCPs’ preferences (about half preferred video), manage logistical considerations (e.g., conducting interviews in multiple practices in the Region on the same day), and account for HCPs’ time constraints. PNS conducted the interviews which lasted 30–60 min and were recorded as audio files for the in-person interviews and as audio-video files with auto transcription for the video interviews. The interviews were semi-structured and guided by the CFIR interview guide provided at www.cfirguide.org. As recommended in the CFIR and in other implementation determinant evaluations [46, 50], we did not include the complete CFIR interview guide, but focused on questions considered relevant for our research question. Through group discussions we chose constructs based on a mix of clinical experience, relevance to our focus on context and the intervention, implementation research experience, and information from the feasibility and intervention adaptation studies [24, 25] leading up to the iTAPP study. Supplementary File 2 features the interview guide. PNS translated the CFIR interview guide to Danish and discussed the translated version with SR, ASN, and JS to ensure correct translation.

To minimize recall bias, we encouraged participants to narrate their experiences concerning their use of the 15-method and concerning their implementation process of the 15-method into everyday work [51]. We followed open-ended questions with prompts or asked the HCPs to provide examples to ensure adequate level of details to understand how potential determinants manifested themselves in the practice. Three research assistants transcribed the interviews verbatim. PNS checked and corrected all auto transcribed material for errors.

Cluster one encompassed six practices, one of which had participated in the feasibility study of the 15-method prior to the iTAPP study which meant that the HCPs in this practice had more experience using the 15-method than personnel in the other five practices. In cluster two, the pattern was the same, with five practices having no previous knowledge or experience of the 15-method before their launch date, and one practice with experience from the feasibility study.

### Data storage

Data were stored on secure serves hosted by the Region of Southern Denmark at Odense Patient data Explorative Network (OPEN) [52] in compliance with the European General Data Protection Regulations.

### Data analysis

#### Coding

We worked in a consensual qualitative research approach [53, 54], which is commonly used with the CFIR because its coding scheme is complex and not well suited for inter-rater reliability testing [50, 55]. PNS conducted all initial coding to the CFIR determinants and created a memo for each practice with a short summary of main findings and reflections from the coding process. An overall memo was also developed for reflections on common themes, insights, and findings across practices and determinants and used for discussions in the research group. We followed the central components in a consensus-bases approach [53] by: (i) using open-ended questions in semi-structured interviews; (ii) ensuring multiple perspectives in the data analysis via team discussions and written correspondences including case memos and summaries of ongoing analyses; (iii) ensuring consensus judgments about the meaning of the data via team discussions and by making sure all authors agreed on the final analysis and results; (iv) having an external auditor to oversee or check the process. In this instance, an external qualitative expert from the research support unit at Odense University Hospital and Region of Southern Denmark [52] without affiliation to the project group; Finally, (v) using cross-oriented analyses, e.g., identify and compare determinants across practices.

We used a directed content analysis for coding, starting from theory and structured with pre-defined codes, i.e. the CFIR determinants [56]. We utilized Nvivo 12 for analysis with a pre-populated template containing CFIR determinants [57] as coding instrument. We created codes for each practice and HCP and linked the HCP codes to their respective practice codes. We adhered to the CFIR codebook and allowed for coding of data to more than one determinant [57]. We further allowed for inductive coding of additional determinants if identified during analysis as presented in other CFIR-guided evaluations [56, 58]. Regarding CFIR domain IV, Individuals, high level leaders referred to the GPs, Implementation Leads referred to all HCPs, Innovation Deliverers referred to all HCPs, and Innovation Recipients referred to patients. Sites did not contain mid-level leaders, formal implementation teams, or “other implementation support”.

All constructs were assessed from the perspective of the HCPs. This meant that aspects such as patient motivation were evaluated based on the providers’ perceptions rather than directly from patients. Additionally, constructs within Domain V, Process, focused solely on implementation process as viewed through the lens of the HCPs.

#### Rating

We rated the valence (positive or negative) and strength (-2 to + 2) of determinants in Domain I, II, III, and V across each practice, following Damschroder and Lowery 2013 [50]. Determinants were rated as neutral (0) if they were mentioned by the interviewees but had no influence on implementation. Determinants were considered to have a weak positive or negative valence (-1/+1) if they were either (i) mentioned in passing or in general terms without provision of details or concrete examples, (ii) if they were associated with both positive and negative comments with an overall positive/negative influence, or (iii) if the determinant was absent, i.e. not mentioned in the interviews, but sufficient data supported an indirect inference about its influence. Determinants with a strong rating (-2/+2) were explicitly described by interviewees, and details or strong statements conveyed key or all aspects of the determinant. We also included a mixed rating category (X) following the rating instructions provided by the CFIR research team [57] for determinants containing contradictory statements, i.e. both positive and negative ratings. Specifically for Domain IV, Individuals, data were analyzed using a matrix with Roles in rows and Characteristics (need, capability, opportunity, motivation) in columns following Damschroder et al. (2022) [29] and assessed for overall patterns using matrix query statistics such as word- and case counts and coding percentages (Supplementary File 3).

We calculated an overall rating of each determinant to assess the overall valence of each determinant in early stages of implementation. The rating was the rounded-off average of ratings across practices, provided that data from HCPs in at least three different practices were available. This minimum threshold was set to mitigate the impact of idiosyncratic responses and to enhance determinant reflectivity through multiple perspectives, aligning with similar CFIR-based studies evaluating determinants of intervention implementation in primary care [55].

Using a matrix coding query, PNS re-read the coded material and provided an initial rating for each practice based on the identified determinants. Blinded to the practices and the initial ratings, KHV independently rated practices on a sub-set of the data providing a second rating with notes for comparison. The research team then discussed disagreements in coding and ratings until consensus was reached.

## Results

We had insufficient data for an overall rating of eight determinants, seven of which contained data that emerged during analysis and were coded inductively, i.e., were not part of our interview guide and selected determinants of focus. Table 2 presents the overall and the practice-specific ratings for determinants in Domain I, II, III, and V. Supplementary File 3 provides verbatim examples and rationales for the overall ratings along with matrix coding statistics regarding Domain IV. The findings below highlight HCPs’ perceptions on the determinants influencing implementation. All participating GPs held managerial positions in their respective practices.

**Table 2:** Determinants influencing the implementation of the 15-method in 12 general practices in the region of Southern Denmark. Ratings of consolidated framework for implementation research (CFIR) determinants

Name	Practice number												
	Overall determinant rating	1	2	3	4	5	6	7	8	9	10	11	12
**I. Innovation Domain** ^**1**^													
Innovation Evidence-Base	**+1**	M	M	M	M	M	0	M	+1	+1	M	M	M
Innovation Relative Advantage	**+2**	+1	0	0	0	+1	+1	+2	+1	0	0	+1	+2
Innovation Adaptability	**+1**	+1	M	0	+1	0	+1	0	M	0	+1	+1	+2
Innovation Complexity (reverse rated)	**Mixed**	+2	0 X	0	-1	-1 X	+1 X	+1	+1 X	+1 X	-2	0 X	0 X
Innovation Design	**Mixed**	-2	+1	-1	+1	-1 X	+1	+1 X	+1	+1 X	-1	0	0
Innovation Cost	**Mixed**	M	0	0	M	M	-1	+1	M	M	M	M	0
**II. Outer Setting domain** ^**2**^													
Local Attitudes	**-1**	M	-1	M	M	M	M	-1	0	-1	-1	-1	-1
Local Conditions	**-1**	M	M	M	-1	M	M	M	M	M	0	M	-2
Partnerships & Connections	**-1**	M	M	-1	M	0	-1	M	M	M	M	M	-1
**III. Inner Setting domain** ^**3**^													
Structural Characteristics ^a^ *Information Technology Infrastructure* ^*a*^ *Work Infrastructure* ^*a*^													
	**-1**	M	-1	M	-1	M	-1	M	M	-1	M	M	M
	**-1**	0	-1	0	-2	-1	M	-1	-1	-1	-2	-1	-1
Communications ^a^	**+1 (mixed)**	+2	M	M	-1	-1	+2	+2	+1	M	M	M	+1
Culture ^a^ *Recipient-Centeredness* ^*a*^ *Deliverer-Centeredness* ^*a*^ *Learning-Centeredness* ^*a*^ *													
	**+1**	+2	M	M	M	M	+1	+1	0	M	0	+2	+1
	**+1 (mixed)**	+1	M	M	-1	-1	+2	+1	M	M	+1	M	M
	**+1**	+2	-	-	-	-	+1	+1	-	-	+1	-	-
Tension for Change ^b^	**+1**	+1	+1	+2	+1	M	+1	+1	+1	+1	+2	M	+1
Compatibility ^b^	**+1**	+2	-1	0	0	0 X	+1	+1	+1 X	+1	M	0	+1
Relative Priority ^b^	**Mixed**	+2	0	0	-1	0	+2	+1	0	-1	+1 X	-1	-1
Incentive Systems ^b^ *	**Mixed**	+1	-	-	0	-	-1	0	-	-	-	-	-2
Access to Knowledge & Information ^b^	**+1 (mixed)**	+2	M	+1	0	0	+2	+1	0	0	-1	-1	M
**V. Implementation Process Domain** ^**4**^													
Teaming	**+1**	+2	+1	M	M	M	+2	+2	M	0	0	M	+1
Assessing Needs *Innovation Deliverers* *Innovation Recipients*													
	**Mixed**	M	-1	M	M	-1	+1	M	M	0	+2	M	M
	**+1**	+1	M	M	M	M	+1	0	M	0	+2	0	M
Assessing Context	**+1 (mixed)**	M	+1	M	0	-1	0	+2	0	+1	0	M	+2
Planning	**-1 (mixed)**	+1	-1	M	-2	-2	+1	M	0	-1	0	-2	M
Tailoring Strategies *	**0**	-	-	-	-	0 X	-	+1	0	0	+1	0	-
Engaging *Innovation Deliverers* *Innovation Recipients*													
	**Mixed**	M	-1	M	M	-1	+2	M	M	0	+1	M	M
	**Mixed**	M	-1	M	M	M	-1	0 X	+1	0	-1	0	-1
Doing	**Mixed**	+2	M	+1	-2	-1	+2	+1	0 X	-1	0	+1	+1
Reflecting & Evaluating *Implementation* *Innovation*													
	**Mixed**	+2	M	-1	-2	-2	+1	0	M	M	+2	+1	+1
	**0**	M	M	M	M	+1	+1	M	M	M	0	0	0
Adapting	**+1**	+1	0	M	M	0	M	+1	M	M	+1	+1	+1

The table presents determinants with available data from ≥3 practices. *Not included in interview guide but included during analysis. M, missing. X, mixed positive and negative statement valence. Strength of valence rages from -2 to +2. ^1^Relates to the “thing” being implemented, i.e. the 15-method. ^2^ The setting in which the Inner Setting exists. In this case, the Region of Southern Denmark and Danish primary health care sector in which the practices exist. ^3^The setting in which the innovation is implemented, i.e. the individual practices. ^4^The actions and/or strategies applied to use and/or implement the innovation, e.g. the degree to which the healthcare professionals teamed up (determinant “teaming”) to coordinate and collaborate on interdependent tasks to implement the innovation. ^a^ Determinant exist regardless of the innovation or the implementation/delivery thereof, the determinant is inherent in the Inner Setting. ^b^ Determinant is specific to the implementation and/or delivery of the innovation

### Barriers to implementing the 15-method

#### Local attitudes (outer setting domain)

Sociocultural values and beliefs regarding “normal” alcohol consumption influenced how easy HCPs found it to ask about alcohol habits. This determinant was closely tied to the perceived *Innovation Complexity*, *Engaging Innovation Recipients* and patients’ motivation and needs (Domain IV):

*“I mean it’s difficult to talk about (alcohol). They (the patients) find it culturally completely acceptable. And we have a lot going on in a consultation. It matters for sure – where peoples’ general mindset is” (HCP 27).*

#### Local conditions (outer setting domain)

This determinant contained little data. HCPs from three practices expressed negative statements regarding the current regional and national set-up for treatment of alcohol problems in general practice, also presented in *Incentive Systems*. Statements included lack of economic compensation to GPs for treating alcohol problems, lack of local specialized treatment facilities, and negative perception regarding the general tempo and busyness of Danish general practice:

*To be honest*,* I don’t know what will happen in general practice – In general*,* not just here – If things keep being so busy. I don’t see how we are supposed to fit anything else in … My colleagues are down to ten-minute consultations. You can barely get the patient through the door and get their blood work done before the time is up” (HCP 10) / “There is definitely a need. The Regions (regional healthcare system) should prioritize this gray-zone of patients with moderate alcohol problems … Targeting this patient group makes sense and I can see how some patients don’t want to go to specialized addiction treatment facilities. They don’t see themselves going there. But we lack dedicated time for them… (HCP 7)*.

#### Partnership and connections (outer setting domain)

This determinant assesses how well the practices are networked with their outer setting, e.g. referrals, organizations and other external entities. The determinant contained little data. HCPs from three practices expressed a low degree of connection to external networks and referral options. The HCPs perceived their number of options in their municipality to be low or unknown, while logistical and patient-related barriers seemed to increase the *Tension for Change*:

*I sometimes have patients who take up a lot of resources*,* and I want to help them*,* so I make time for them. I know I can refer them to the specialized treatment facility but it’s just too far for us out here. And the municipality – it’s hard to figure out what they actually offer (for this patient group) so I figured it would be nice if I could do something extra*,* to get some additional tools myself and maybe do something about it myself” (HCP 9) / “We already get most of our referrals (to other specialists) rejected and there is going to be a big fight for our resources (in general practice). (HCP 7)*.

#### Structural characteristics (inner setting domain)

We found two sub-determinants related to *Structural characteristics* in the inner setting that were identified as barriers:

#### Information technology-infrastructure (structural characteristics, inner setting domain)

The practices’ IT systems differed which meant that the HCPs had different opportunities for using their IT system to support their daily work, e.g. the use of statistics and ease of data retrieval from the system. Regarding digital solutions and technology infrastructure, our data suggested that the HCPs wanted more digital solutions for the 15-method, e.g. a digital version of the AUDIT, and were motivated by for example statistics and visual representations of their progress using the method.

#### Work infrastructure (structural characteristics, inner setting domain)

This determinant includes organization of tasks, responsibilities between HCPs, staff availability, workload, and time availability. Time and staff were the most frequent barriers within this determinant. HCPs frequently cited lack of time in their everyday work as a barrier to implementation. Specifically, they reported insufficient time for implementation efforts such as maintaining the new practice, reflecting on, or organizing the work. Busy schedules and long waiting times were frequently brought up as barriers to making time for implementation efforts:

*… It’s just way too busy here. Both of my colleagues quit*,* and I just can’t keep up. So I haven’t handed out anything other than one of those flyers… I think it’s a shame… It’s just too busy*,* we have way too much to do because we are understaffed (HCP 10) /*.

*… from a resource point of view*,* we have not been able to make time in our schedule for supervision. Right now we have up to eight weeks waiting time for our patients… we sometimes have to schedule things in late afternoon in our overtime… logistically it’s very difficult (HCP 26)*.

#### Planning (implementation process domain)

This determinant focuses on identifying roles, responsibilities, and defining goals, measures and milestones for implementation. It had an overall negative mixed score. No HCPs defined goals or measures for implementation success. HCPs in two practices presented clear roles and responsibilities for the delivery of the intervention, while the HCPs in the remaining practices either had no explicit plans, lacked clear responsibilities, or had not communicated among staff groups how to use the intervention in their practice. This determinant was related to the *Communication* in the practice; less frequent or inefficient communication seemed to be associated with a lower degree of planning, and vice versa.

### Facilitators for implementing the 15-method

#### Innovation evidence-base (innovation domain)

HCPs perceived training in the 15-method and the method’s material to be credible and based on trustworthy evidence. Some HCPs became more aware of pharmacological treatment options through training in the 15-method, and others found the evidence concerning symptom-based screening to facilitate their use of the method.

*After listening to your session on pharmacological treatment*,* I thought a lot about it*,* because we had a number of patients who were on Disulfiram. After seeing the data you showed us I started a patient on Campral instead*,* while having your session in the back of my mind (HCP 24)*.

#### Innovation relative advantage (Innovation Domain)

This determinant evaluates the degree to which HCPs perceive the 15-method to be better than other available innovations or current practice. We found that the HCPs were positive towards the 15-method, highlighting its structured approach and support in discussing alcohol with tangible and structured next steps. Some HCPs struggled to find the time to use the method in a busy consultation, which tied this determinant closely to *Work Infrastructure* (described below).

*I think it’s great that we now have a tool to help us go more in depth. I am still surprised at how many drink more than they are ‘allowed’. But now it’s something we can get a hold of and ask “would you like to talk about this?” before*,* I would just have said ‘you can’t drink that much; you should stay below the national recommendations’ (HCP21)*.

#### Innovation adaptability (innovation domain)

This determinant assesses the degree to which the 15-method could be modified or refined to fit the context. Most of the HCPs found that the mix-and-match structure and the possibility for interdisciplinary work with 15-method made implementation easier. HCPs in several practices had ideas for tailoring the method to their specific practice e.g., who in the practice could conduct screening and how to make sure patients had flexible follow-ups.

#### Communications (inner setting domain)

This Inner Setting determinant assesses the degree of information sharing within the practice. It had a mixed but overall facilitating influence. Practices with a high degree of formal and informal communication used the intervention more and the staff expressed higher levels of comfort using the 15-method. In practices with positive ratings, HCPs were more likely to have adjusted their strategies for implementing the 15-method, and were more likely to reflect on their progress, as illustrated in the following example:

*We have an ongoing evaluation*,* and I think it is possible because we are a small clinic. We have a thirty-minute coffee break every morning and we always eat lunch together. That makes time for us to talk about even the smallest things*,* and we usually discuss professional topics. On top of that we have our scheduled staff meetings every four weeks to keep us ahead in our planning. (HCP 1)*.

In practices with sparse communication, the scores in the determinants *Planning* and *Reflection and Evaluation* were lower, and staff were more likely to find the 15-method complex and less compatible to their work. As one HCP said on communication in their practice regarding planning and reflection:

*Everyone just started on their own and tried to use it (the 15-method) the best they could. I think we might have brought it up once or twice but what we actually discussed… well… I think it was just randomly brought up during lunch one day. (HCP 11)*.

#### Culture (inner setting domain)

This determinant assesses the degree to which there are shared values, beliefs, and norms around “caring, supporting, and addressing the needs and welfare of recipients/deliverers” or around “psychological safety, continual improvement, and using data to inform practice” [57]. The determinant contains four sub-determinants, three of which we found to facilitate implementation.

##### Recipient-Centeredness (culture, inner setting domain)

HCPs in practices with higher recipient-centeredness, were more flexible in their approach to using the elements of the 15-method and to accommodate for their patients’ resources, needs, or motivation. The following example illustrates how a GP used the 15-method as an adaptable approach, to ensure a patient-centered progress:

*He (the patient) drinks less now*,* he feels better*,* and when I ask him what his goals are he is like ‘well*,* if you tell me it’s unhealthy to drink that much I’ll try to reduce my intake’*,* and that’s what we’re working towards right now. But I’m really trying to make his motivation carry this forward - that’s sort of the point right. I’m not supposed to sit here and pretend to be clever (HCP 7)*.

##### Deliverer-centeredness (culture, inner setting domain)

This had a mixed but overall positive rating. We found that this determinant was closely tied to *Communications* and *Work Infrastructure*, as staff in practices with lower deliverer-centeredness generally had a perception of low resources and/or less communication in their practice. As one staff member stated it in a practice with a culture of high deliverer-centeredness:

*Us nurses are always the ones writing up our new checklists and such in our patient filing system. It takes a lot of time*,* but we always have the freedom to just make the time in our schedule. It might take a couple of hours but it’s never a problem. If we aren’t finished by that time*,* we just schedule another timeslot. He (the practice owner) knows we need the time*,* and he knows one needs the time to make something happen (HCP15)*.

##### Learning-centeredness (culture, inner setting domain)

HCPs in three practices stated that learning and getting better at something had influenced their decision to participate in the iTAPP project. HCPs in two practices also stated that they prioritized participating in projects, such as the iTAPP study, to be able to contribute to research in addition to their own learning.

The fourth sub-determinant within *Culture*, human equality-centeredness, was not included in our interview guide and did not include any data.

#### Tension for change (inner setting domain)

This determinant assesses the degree to which the current situation is (in)tolerable and needs to change. It varied between HCPs as to what they perceived needed to change. One common perspective was that the way in which alcohol was addressed in the practice could be better, both in terms of focus, approach, and priority. Some HCPs felt that the stigma surrounding alcohol needed to be addressed, as it posed a barrier for both providers and patients. This stigma hindered HCPs from delivering care to the best of their abilities and limited open conversations with patients. Another general perspective was that treatment of alcohol problems was fragmented between sectors and the HCPs would need a concrete way to go about treatment in their practice e.g., specific treatment material as in the 15-method. Several HCPs also pointed to the fact that they had a hard time referring their patients to specialized treatment facilities since many of the treatment facilities were located far away from the general practice. These tensions helped facilitate implementation of the 15-method because the HCPs wanted options and tools different from their current situation as illustrated in the following:

*… when we saw your project*,* I thought ‘that’s a topic I don’t discuss enough with my patients’ and it is exactly that - the barriers surrounding alcohol. Are those barriers with me or the patients… We as GPs have a responsibility to ask about such things*,* it’s an aspect of overall health and I got curious whether we could get some new tools or routines to get better at it” (HCP2) / “It would be great if we can keep it going and learn something from this project*,* because the nearest treatment facility is far away and many of our patients don’t even have a car and driving to alcohol treatment - well that’s in many cases just a bad idea. We need something closer. (HCP 26)*.

#### Compatibility (inner setting domain)

This determinant assesses to what degree the HCPs perceive the 15-method to fit with their workflows, systems, and processes. This was closely tied to *Relative Priority* and *Innovation Complexity*, as HCPs who perceived the 15-method to be too complex found the method to have low compatibility with their work and were less likely to prioritize resources for implementing the 15-method. However, most of the HCPs found the 15-method compatible, as illustrated in the following statements:

*We have incorporated it (the 15-method) into many of our daily routines and conversations regarding alcohol. I don’t think it takes up extra time any longer” (HCP 2). / “I have always asked about lifestyle factors and now when I think ‘well in this case there might be something more’ I have this (the 15-method) to use*,* right? (HCP 9).*

#### Access to knowledge and information (inner setting domain)

This determinant assesses the degree to which the HCPs felt they had sufficient training and guidance to implement the 15-method, and to what degree they felt comfortable using the project website and material. The determinant had a positive but mixed score. Most of the HCPs were positive or neutral as to whether they felt training in the 15-method had been adequate for them to use the intervention and whether they felt comfortable with where to find material and support. HCPs from two practices stated that they found their staff needed longer training sessions and more hands-on training to be comfortable delivering the intervention:

*I think I can apply it (the 15-method)*,* I just took what I found available… I don’t know my way around all the material yet*,* but I use what I know and when I feel like it*,* I add some more” (HCP 22). / “I have visited the website a couple of times to peek around. Just to get comfortable with it and know where to look if I want to print something during a consultation (HCP 17)*.

#### Teaming (implementation process domain)

This determinant concerns the degree to which HCPs team up, coordinate, and collaborate to implement the 15-method. This was closely tied to the degree of formal and informal communication in the practice, and the degree to which task responsibilities were clear. In practices with a high degree of teaming, the leadership was clear in delegation of tasks, and staff had time to coordinate and discuss implementation of the 15-method.

#### Assessing Needs *- innovation recipients* (implementation process domain)

This determinant focuses on the degree to which the HCPs assessed the priorities, needs, and preferences of their patients and included data from five practices. In all five, the HCPs expressed an inherent patient-centered approach. The HCPs were attuned to listening to patient priorities, preferences, and needs. Assessing recipient needs was closely tied to the determinant *Compatibility* and patient motivation and needs as the HCPs found it challenging to use the 15-method’s material or “getting to” questions on alcohol habits if the patient had specific priorities or preferences to the content of the consultation different from the topic of alcohol habits. Assessing patients’ needs facilitated implementation as it entailed the HCP and patient worked together on the patient’s needs, including elements from the 15-method when relevant, which also enhanced the perceived compatibility of the 15-method.

#### Assessing context (implementation process domain)

Similar to assessing needs, this determinant assesses the degree to which HCPs collect information about their context to identify and address facilitators and barriers for implementation. This determinant had a positive mixed rating and was tied to the determinant *Local Attitudes* regarding barriers for addressing alcohol. The HCPs were generally good at identifying barriers and facilitators in their context to implement and deliver the 15-method. The barriers were predominantly related to alcohol culture, stigma, and fear of damaging the patient-provider relationship:

*If the patients get a question on alcohol a lot*,* they learn that*,* just like tobacco*,* it’s just part of the conversation and we ask them. That might help*,* but right now we have to be careful. The change has to occur out there*,* in the real world outside our walls. It’s definitely a challenge for us*,* because it demands that the patients accept that there is a problem… but right now it is socially acceptable*,* and we can’t change that. It takes time. (HCP 4).*

#### Adapting (implementation process domain)

The HCPs were attentive to how the 15-method could be adapted or modified to fit their practice, patients, or work infrastructure. HCPs in practices with a neutral rating had considered how possible modifications might occur or envisioned future use of the intervention.

### Determinants with neutral influence on implementing the 15-method

#### Tailoring strategies (Implementation process Domain)

This determinant assesses the degree to which HCPs selected or conducted strategies to address barriers, fit their context or use facilitators to their advantage. No HCPs had made an explicit implementation plan or strategy at the time of data collection. HCPs in four practices had either made or considered taking small measures to facilitate easier application of the 15-method’s material and support conversations on alcohol, e.g. where to place the material for ease of use and incorporate phrases and communication tips into their IT patient filing system.

#### Reflecting and evaluating – *innovation* (implementation process domain)

The rating refers to whether and to what degree the HCPs reflected on and discussed the effectiveness of the 15-method. This determinant was neutral, and the HCPs had made no formal data collection on this matter. Overall, the HCPs had reflected little on their perceived success of the 15-method prior to the interviews and had only to a limited degree discussed the success of the method.

### Determinants with mixed influence on implementing the 15-method

#### Recipient needs and motivation vs. deliverer opportunities and capabilities (individuals domain)

The tension between patient needs and motivation and HCPs’ opportunities and capabilities in delivering the 15-method played a key role in all interviews. HCPs’ generally perceived patient motivation for discussing alcohol as low. Additionally, patients often prioritized addressing immediate concerns over long-term risks and the interaction was further complicated by variations in HCPs’ self-perceived capability in delivering the 15-method. While most HCPs were motivated and capable, they found it challenging to navigate a sensitive topic within time constraints especially when they perceived patient motivation to be low or perceived the intervention to not meet the patient’s current needs for the specific consultation. Barriers such as stigma, fear of offending the patient, or difficulty assessing patient readiness, could lead HCPs to feel underprepared or in need of further training. Contrary, a positive synergy occurred when patient needs and motivation were high, whether due to a clear recognition of the problem, symptom connection, or interest in related treatments (e.g., weight loss medication), and when HCPs’ capabilities aligned with those needs. HCPs opportunities to deliver the 15-method were influenced by determinants such as structural characteristics, innovation relative priority, and assessing the needs of the intervention deliverers. The following three quotes illustrate contrasting perspectives on HCPs’ own capabilities and opportunities to deliver the 15-method. While some felt confident even when having limited opportunities to deliver the intervention, others felt constrained by either their own abilities or their opportunities. Low patient motivation further complicated this dynamic and affected some HCPs more than others:

*I don’t have to sit here and convince (the patient). I don’t have to fight either. The patient is here out of his own will*,* and by being a catalyst I can help the process along.” (HCP 7) / “I think we experience more and more how much of a taboo it (alcohol) really is. People may see that they drink too much*,* but then say*,* “and that’s not a problem for me” and then the next step*,* to say that it might be a problem – to convince them*,* I find that really difficult… Perhaps we are just not skilled enough.” (HCP14) / “We can talk about it*,* but we can’t shift the agenda enough to motivate the patient. They often have a need for treatment*,* but they don’t have any motivation for treatment. It just doesn’t move (them) enough – they might say they’ll think about reducing their drinking*,* but I can’t get anyone into anything concrete regarding the 15-method. They slip away from us (HCP 16)*.

#### Innovation complexity (innovation domain)

This determinant assesses how complex the HCPs perceived the intervention to be and it is reverse-rated, i.e. a negative rating indicates more complexity. Most of the HCPs found the steps in the 15-method to be simple and the structure easy to understand. They did, however, also find the material to be too extensive and occasionally too complicated. The most prominent factors related to the perceived innovation complexity were HCPs opportunities to deliver the intervention (e.g., time constraints) and patient needs and motivation to talk about alcohol or change alcohol habits. The HCPs who found the method more complex relative to their peers, found the method’s “fixed” structure unsuited for the complexity of a messy consultation, found it difficult to determine what material to use when, and how to use the method when facing low patient motivation. The following quotes illustrate the mixed perception of the 15-method’s complexity:

*The tools are fine*,* but the biggest challenge for me is time. With the 15 minutes I have for a full yearly check-up*,* I mean - there is so much other stuff… The challenge is that the patient comes here because of something else… the patient has to find it relevant to fill out an AUDIT questionnaire” (HCP 25) / “It’s great. I just took what made sense to me… I have worked with motivational interviewing before*,* and I am always trying to stay curious. I think I will use it (the 15-method) more and more along the way (HCP 22)*.

#### Innovation design (innovation domain)

This determinant concerns how well the innovation is designed, packaged, assembled, bundled, and presented. Some HCPs found the material and design of the 15-method to be fun and engaging, also for their patients, while some HCPs found the layout of the patient material to be unserious. Generally, the HCPs stated that the patient material was overly complex and/or excessively extensive. “Icebreakers” such as posters, mugs, and bottles, were received positively and neutrally. Not all practices used these items. The following quotes illustrate the mixed perceptions of the material among HCPs:

*It can be a sensitive topic and if one chooses to do something about their alcohol habits the material has to be serious as well… The illustrations and drawings - we just thought… you know*,* they’re silly or not serious*,* in some way.” (HCP 2) / “Did you see we put the posters up? And the bottles are great… and we have the flyers lying around in the waiting room and you can just see how they (the patients) are fiddling with them and reading them. (HCP 21)*.

#### Innovation cost (innovation domain)

This determinant assesses the degree to which operating costs and purchase of the intervention are affordable, but as HCPs were compensated for their time spent in the iTAPP study, they had no explicit monetary operating cost. Some HCPs worried that they, beyond the scope of the iTAPP study, could generate more “alcohol-related” consultations in their practice for which they would not be compensated. Other HCPs did not share this view and saw “alcohol treatment” as within the scope of their work, why this determinant had a mixed rating. This aspect is elaborated in *Inventive Systems* below.

#### Relative priority (inner setting domain)

This determinant assesses how important the HCPs find the implementation and delivery of the 15-method compared to other initiatives. We found that this was tied to how compatible the HCPs considered the method to be (*Compatibility)*, how complex they viewed the intervention to be (*Intervention Complexity)*, and how many available resources they had to implement the intervention (*Work Infrastructure*). Low resources regarding time and staff lowered the relative priority, while shared motivation or focus regarding alcohol issues in the practice increased priority. As one GP expressed the many aspects of making something a priority:

*We don’t have any other major projects going on right now*,* but we are challenged due to high levels of sick leave among our staff at the moment… I mean*,* it’s no excuse*,* I guess that’s just how it always is… This (addressing alcohol) is something I get excited about*,* but it’s difficult - we all think it’s relevant*,* it’s just hard to figure out how we can fit it in and keep it going (HCP 24)*.

#### Incentive systems (inner setting domain)

This determinant assesses how different incentives affected the implementation of the 15-method. Data from five practices indicated that the HCPs who were incentivized to deliver the intervention had personal interest in the topic or stated that they believed the intervention could improve the treatment of their patients. No HCP perceived the economic compensation for participating in the iTAPP project as a reward or incentive to implement the 15-method. A few HCPs stated that they were disincentivized to continue the 15-method intervention after the iTAPP project due to the current national economic structure for treating alcohol related issues in general practice which does not include separate reimbursement to the GPs for treating alcohol specific issues:

*I hear many stories from our patients. They (alcohol problems) are present in almost any family… But patients with addictions are in a group that some people just distance themselves from… someone needs to be the front-runners. I want to support this (project)” (HCP 1). / “I can imagine it (money) could be a problem; if we for instance treat a patient with diabetes who also has an alcohol problem – we would not get paid (for treating the alcohol problem). So it will become a problem if we are going to have many extra consultations because we are not getting paid to do alcohol treatment (HCP 15)*.

#### Assessing needs – *innovation deliverers* (implementation process domain)

The degree to which GPs in managerial positions communicated with their staff and collected information on the needs of the innovation deliverers varied significantly between practices. In some instances, HCPs, including managerial GPs, had shared responsibilities regarding the delivery of the intervention which entailed collective reflection on their priorities and thus a high degree of teaming. HCPs in practices with a negative score stated that their needs and preferences were not heard and/or questioned the backing and support from their managers.

#### Engaging (implementation process domain)

This determinant holds two sub-determinants assessing (i) the degree to which HCPs encourage other staff members (deliverers) to implement the 15-method, and (ii) the degree to which HCPs attract patients (recipients) to use the 15-method.

##### Innovation deliverers (engaging, implementation process domain)

Practices with frequent informal communication on implementation efforts were more likely to engage the intervention deliverers (HCPs) as their communication facilitated ongoing adjustments and kept the team engaged in trying out or using the intervention. A common aspect among practices with low deliverer engagement was a lack of specified time to work through, supervise, and plan how to use the intervention in their practice, which relates to *Relative Priority*,* Communication*, and *Work Infrastructure*.

##### Innovation recipients (engaging, implementation process domain)

We focused this determinant on the HCPs’ perception of their patients’ level of engagement and use of the 15-method material. Overall, the HCPs stated that they had difficulties engaging their patients in using the 15-method’s material and moving from screening to treatment (from step 1 to step 2 and/or 3). The HCPs related this challenge to the existing alcohol culture in Denmark as well as to stigma around alcohol problems. Engagement of recipients was further influenced by the HCPs’ (self-perceived) communicative skills, comfort level with addressing alcohol problems, available time in the consultation (*Work Infrastructure* and *Opportunity*), and the challenge of engaging the patients in a conversation on alcohol if the patient had a different agenda, different needs, or low motivation.

#### Doing (implementation process domain)

This determinant assesses the degree to which the HCPs implemented or tested the 15-method in small steps or cycles to optimize delivery. We found great variation in the degree to which HCPs had tested and tried the 15-method material and its integration into their daily routines. Responses varied from no use of the material or method at all, to high degrees of structure, planning, and trial cycles of material and staff involvement to provide internal feedback in the practice. The following quotes present first a response from a practice with a high degree of *Doing* and a second with low degree:

*In the beginning we used it (AUDIT screening) in every cardio-vascular control. We now use it in COPD (chronic obstructive pulmonary disease)*,* diabetes*,* and osteoporosis controls as well… My nurse handles some of the yearly controls. If she is the one doing the screening*,* she is also the one doing the follow up consultation. Previously*,* I did all the yearly controls*,* but we thought it more natural for her to do the follow-ups and take some of these conversations. I mean*,* we’re currently testing it.” (HCP 2) / “I think I’m more aware of alcohol related problems now*,* but I haven’t really had a patient who I thought might have a problem… I think I have handed one (AUDIT questionnaire) out*,* and I think the patient was to be scheduled for a follow-up with our GP*,* but I haven’t heard anything (HCP 25)*.

#### Reflecting and evaluating - *implementation* (implementation process domain)

This determinant assesses the degree to which HCPs collected and discussed information about the progress of the implementation. HCPs in practices with high frequent informal and formal communication (*Communication*) were more likely to discuss and reflect on the implementation process in their practice. HCPs in practices with a negative score had little or no ongoing evaluation or communication concerning their implementation process, had no actual ongoing implementation efforts, or stated that they had not had the time to make such plans or evaluate the process.

## Discussion

The present study aimed to assess healthcare professionals’ perceptions of determinants for early-stage implementation of the 15-method in Danish general practice and to classify these determinants using the Consolidated Framework for Implementation Research (CFIR).

From the HCPs’ perspective, the most important barrier was the tension between patient needs and motivation and provider opportunities to use the 15-method, complicated by HCP’s self-perceived capabilities. Being short on time, perceiving a low patient motivation, or feeling inadequately equipped to discuss alcohol in depth all hindered HCPs’ use and increased the perceived complexity of the 15-method. Further, implementation was hindered by low attention to staff members’ needs related to delivering the intervention, emphasizing the need for adequate opportunities to deliver an intervention [49, 59].

HCPs identified several factors that facilitated the implementation of the 15-method in general practice. The method’s adaptability and compatibility with existing practices, coupled with its strong evidence base, and relative advantage over current practices, including easy access to knowledge and information, facilitated implementation. Further, effective communication within practices, a perceived tension for change, and a practice culture that emphasized learning while prioritizing staff and patient needs supported implementation.

Nearly one-third of the identified determinants had mixed ratings, highlighting the complexity of implementing an intervention across 21 different practices which may differ in many contextual aspects [60]. What we identified as a facilitating determinant in one practice could act as barrier in another (Table 2), underscoring the significant influence of local context. On example is the degree to which the HCPs accepted the “new behavior” of the 15-method [61]. The acceptance of change in a team or workplace is dependent on the culture and leadership. Leaders are often considered important “culture creators” because of their power to influence group members with their norms and values, but culture also influences who is recognized as leader [62]. We found that the leaders of implementation for this “new behavior” were not necessarily the GPs. Instead, nurses sometimes functioned as implementation champions for the method. Implementation champions are individuals who are internal to the implementation setting and committed to implementing a change, often without formal compensation, and can help drive implementation through diverse challenges such as low stakeholder engagement by persistence or their strength of conviction in the intervention [63]. The use of implementation champions is a commonly recognized approach when designing implementation strategies [50], albeit not a planned aspect of the iTAPP study.

HCPs found the 15-method more complex when faced with communicative challenges, such as fear of stigmatizing the patient, particularly under time constraints, or when the patient’s agenda was different from that of discussing potential alcohol-related issues. This perception among HCPs was exacerbated by perceived low patient motivation. While most HCPs stated that they screened more patients for alcohol problems and were more attentive to possible alcohol-related problems, they also stated that they had very few patients who wished to engage in further treatment or in follow-up consultations. The initial screening questions on alcohol were considered feasible, as most of the HCPs felt comfortable asking routine questions. However, the subsequent steps of the method appeared to be more challenging as the HCPs tried to engage patients in follow-ups or elaborate on the situation. Similar findings of higher screening rates but restricted treatment engagement have been seen in other implementation intervention studies for alcohol treatment in primary care [64] and this combination of high screening and low treatment engagement is a common challenge in alcohol use disorder treatment [65]. It is worth noting that the 15-method in a general practice context with non-alcohol-treatment seeking patients could be considered both a prevention program and a treatment intervention. The overall public health may benefit from higher rates of screening and brief advice [4], but the HCPs’ perception of the intervention may change, e.g. seen as more complex or less useful [47], if the HCPs expect the intervention to be a treatment intervention and experience that few patients return. This could yield lower HCPs acceptance of the 15-method [66] which can negatively influence intervention fidelity, adoption, and sustainability [11, 67]. Thus, in a general practice context it may be beneficial to consider the 15-method to be two interventions in one: asking about alcohol, i.e. screening, which HCPs are generally comfortable with [68], and discussing and treating alcohol problems, which may require more training, skills, and resources than screening alone [16, 69].

Regarding financial incentives, the HCPs received compensation for their participation in both the iTAPP study and the present study. Notably, Danish GPs are not specifically compensated for addressing alcohol-related issues at the time of the present study. We identified two determinants influenced by the economic aspects of current national and regional GP compensation structures: *Innovation Cost* (mixed rating) and *Local Conditions* (-1). As this evaluation occurred within the context of the iTAPP study, the financial incentive structure for the delivery of alcohol treatment did not reflect real-world conditions. Although the participants generally perceived the financial incentive to be of little influence, some HCPs did raise concern that neglect of such incentives could negatively affect future large-scale implementation efforts of the 15-method.

The present study provides an assessment of determinants for early-stage implementation based on healthcare professionals’ perceptions while actively using the intervention. These insights are critical for anticipating potential implementation challenges and informing future strategy development. However, given the absence of formal implementation outcome measures in this study, these findings should be interpreted as generating hypotheses for future evaluation. For instance, the tension between low patient motivation and low provider opportunity to deliver the intervention suggests that monitoring fidelity and adoption rates in later trials will be important for understanding the actual impact of this determinant [70, 71]. Whether the identified determinants align with and to what degree they influence actual implementation outcomes and intervention effectiveness in the iTAPP study is the subject for planned studies, ultimately helping to inform future tailored and theory-based implementation strategies [67, 72]. The variation in determinants across the practices indicate that a one-size-fits-all-strategy for implementing the 15-method in Danish general practice would likely be inefficient compared to a more comprehensive, multifaceted strategy [73, 74]. The Expert Recommendations for Implementing Change (ERIC) project [75, 76] identified 73 implementation strategies, making it possible to select evidence-based strategies that can be aligned with identified barriers although the process of matching specific determinants to appropriate strategies can be quite challenging [77]. Future efforts to implement the 15-method may benefit from exploring what implementation strategies according to the ERIC compilation might be most relevant for facilitating the implementation. It may also be beneficial to guide the implementation by developing a process and/or logic model, clearly specifying implementation outcomes to monitor and evaluate the progress effectively [28, 78, 79]. Potentially relevant strategies from the ERIC compilation to address barriers and mixed determinants identified within CFIR Domain I, II, III and V in this study are provided in Supplementary File 4 while we recognize the need for robust quantitative measures of implementation outcomes and their correlation to the identified determinants before specific strategies can be proposed [50, 80]. Notably, the ERIC tool does not map to the updated CFIR regarding Individual Characteristics (Domain IV) which played a key role in our study. Thus, future strategies should also consider how to improve HCPs capabilities (such as through training) and opportunities to deliver the 15-method, drawing from established theories of behavior change and implementation such as the COM-B model or other role-specific theories and implementation frameworks.

The present study has important limitations. First, as the MRC guidance on process evaluation states, working with intervention stakeholders can be challenging as to whether the researchers should communicate emerging findings to provide feedback and help correct implementation problems or challenges, or merely be passive observers [28]. A more active role is generally found in feasibility testing, whereas effectiveness evaluation is preferably conducted without interference from the researcher, as this might compromise the external validity of the evaluation. In the present study, the evaluators (PNS, KHV) were also part of the iTAPP research team. This requires the evaluators to choose between multiple possible roles during different stages of the project. However, being on the “inside” during evaluation offers distinct advantages. An internal evaluator possesses in-depth contextual knowledge, understanding both the environment and the intervention in detail. This allows for a more nuanced, context-specific evaluation, capturing subtleties that might be overlooked by external evaluators [81]. Additionally, we aimed to include a minimum of one GP and one nurse in each practice to provide multi-disciplinary perspectives. In four of the twelve practices, we did not manage to include the nurses’ perspective and in one practice no GP was interviewed. This happened due to logistical constraints in the practices as interviews were conducted during opening hours and a minority of practices were unable to free up staff from patient consultations. This entails that most participants were GPs (17 of 28) who represented both an intervention deliverer and a managerial perspective. Lastly, we focused solely on HCP’s perceptions of determinants, including aspects such as implementation process in their own practices and patient motivation. Future planned studies will address implementation evaluation from the perspective of the iTAPP research team and evaluation from the patients’ perspective.

## Conclusion

Healthcare professionals identified several determinants facilitating early-stage implementation of the 15-method in Danish general practice, indicating that implementation of the method is possible. From the healthcare professionals’ perspective, the main challenge was balancing patient motivation with their own opportunities and capability to deliver the intervention. The findings suggest that a multifaceted implementation strategy may be necessary to address the variations in context and resources across different practices.

## Electronic supplementary material

Below is the link to the electronic supplementary material.

Supplementary Material 1

Supplementary Material 2

Supplementary Material 3

Supplementary Material 4

## Data Availability

No datasets were generated or analysed during the current study.

## Abbreviations

SBIRT: Screening, Brief Intervention, and Referral to Treatment
HCP: Healthcare Professional
AUDIT: Alcohol Use Disorder Identification Test
iTAPP: Identification and Treatment of Alcohol Problems in Primary Care
MRC: Medical Research Council
CFIR: Consolidated Framework for Implementation Research
GP: General Practitioner
ALAT: Alanin-Amino Tranferase
GGT: Gamma-Glutamyl Transferase
ICD-10: International Classification of Diseases 10th revision
OPEN: Odense Patient data Explorative Network
ERIC: Expert Recommendations for Implementing Change
