# Supplementary material for: Determinants of implementing the 15-method in Danish general practice using the consolidated framework for implementation science
Source: Addict Sci Clin Pract. 2025 May 16;20:43. doi: 10.1186/s13722-025-00571-0 (PMC12083036; doi:10.1186/s13722-025-00571-0)
Supplement: Supplementary file 1 — Supplementary Material 1 [file 13722_2025_571_MOESM1_ESM.docx]

The 15-method included posters, bottles, and flyers as icebreakers. The healthcare professionals could use the icebreakers as they preferred.

**Example of posters:**


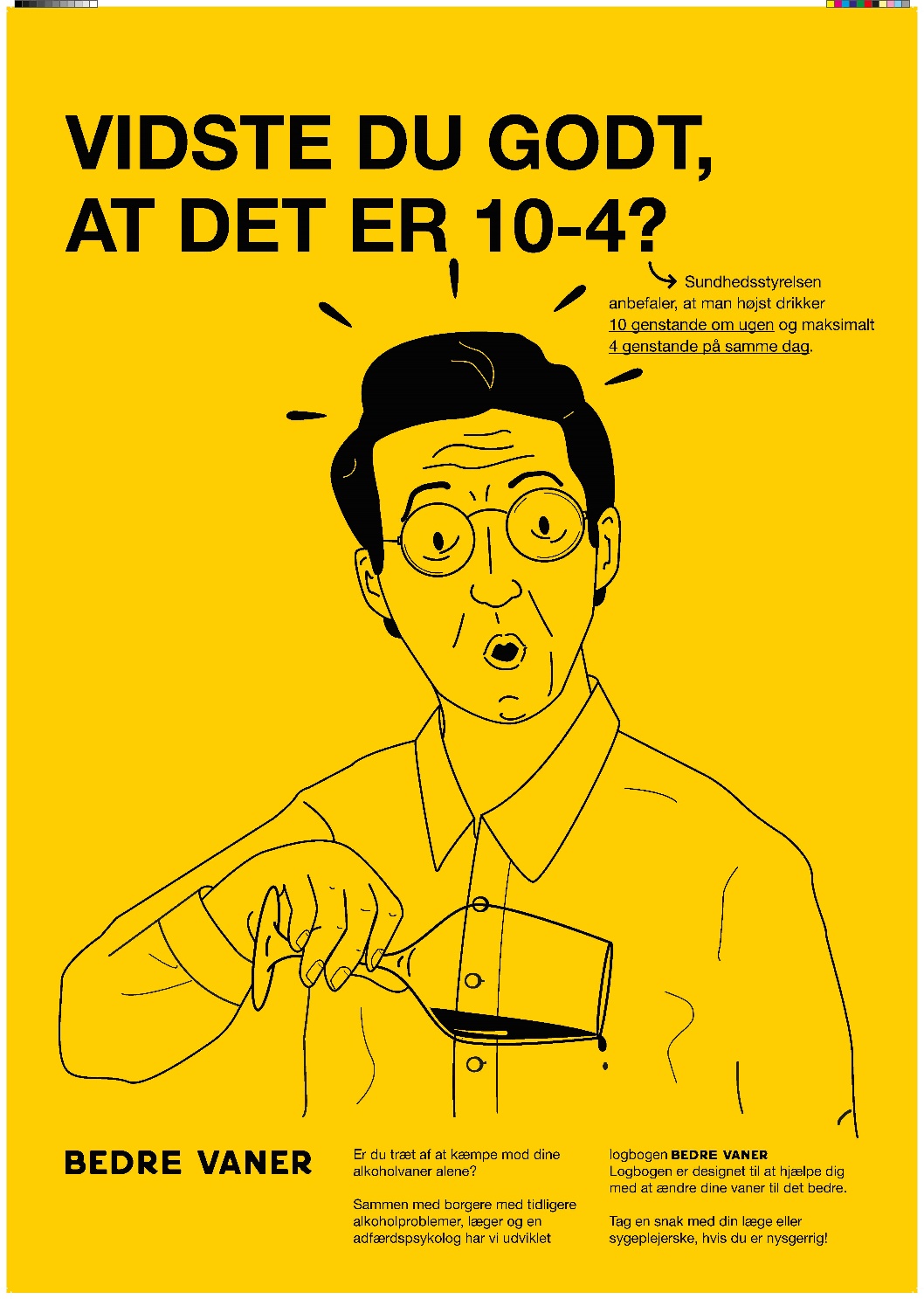


Translated text: (top) ”Did you know it’s 10-4? The Danish Health Authorities recommend a maximum of 10 drinks per week and maximum of 4 drinks in one day.” (bottom) “Tired of battling your alcohol habits on your own? We created the BETTER HABITS logbook with input from citizens with prior alcohol problems, doctors, nurses, and a behavioral psychologist. It aims to help you improve your alcohol habits. If you’re interested, talk to your nurse or doctor.”

Example of posters in a general practice waiting room.


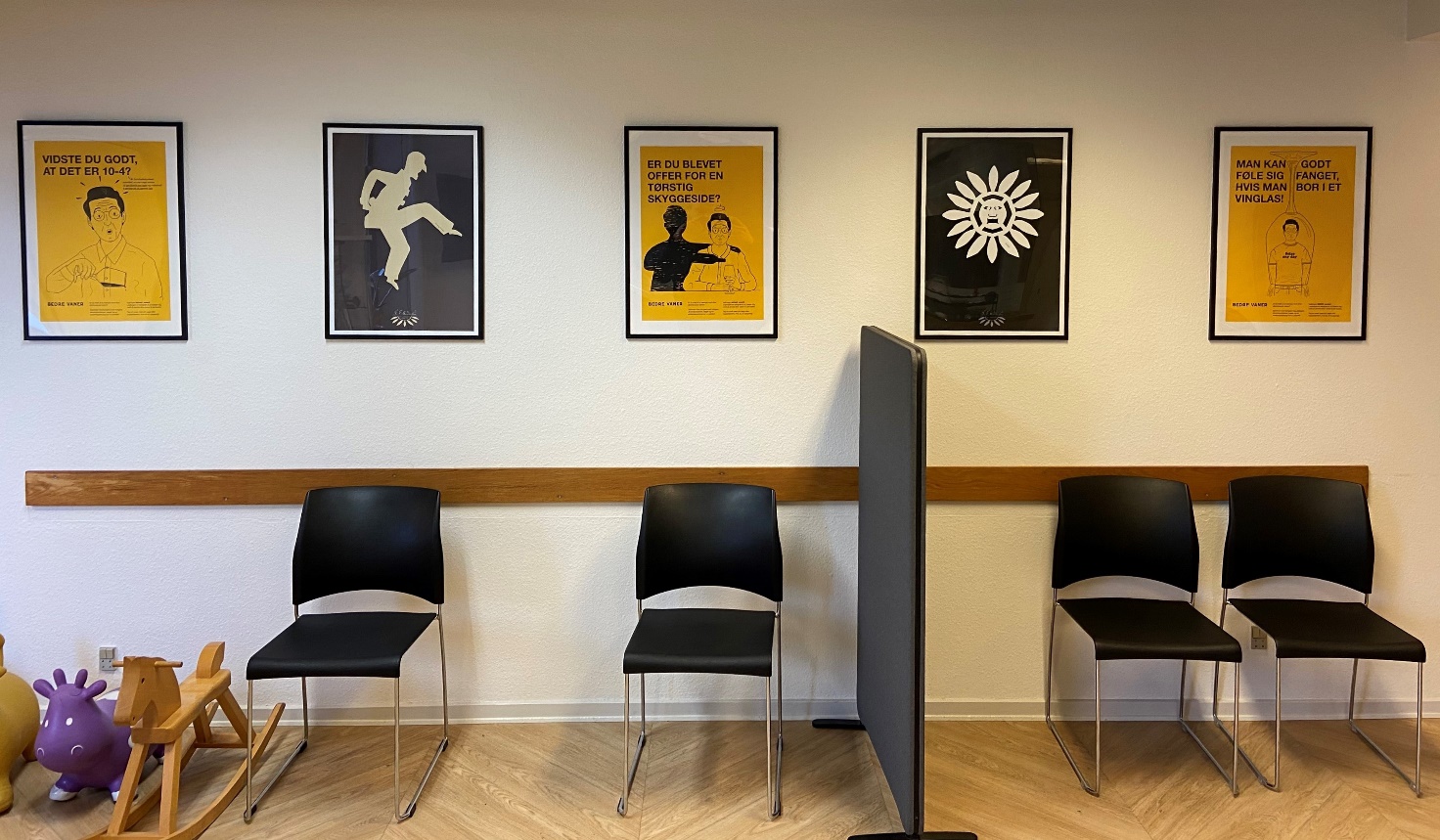


**Example of flyers:**

Front example 1.


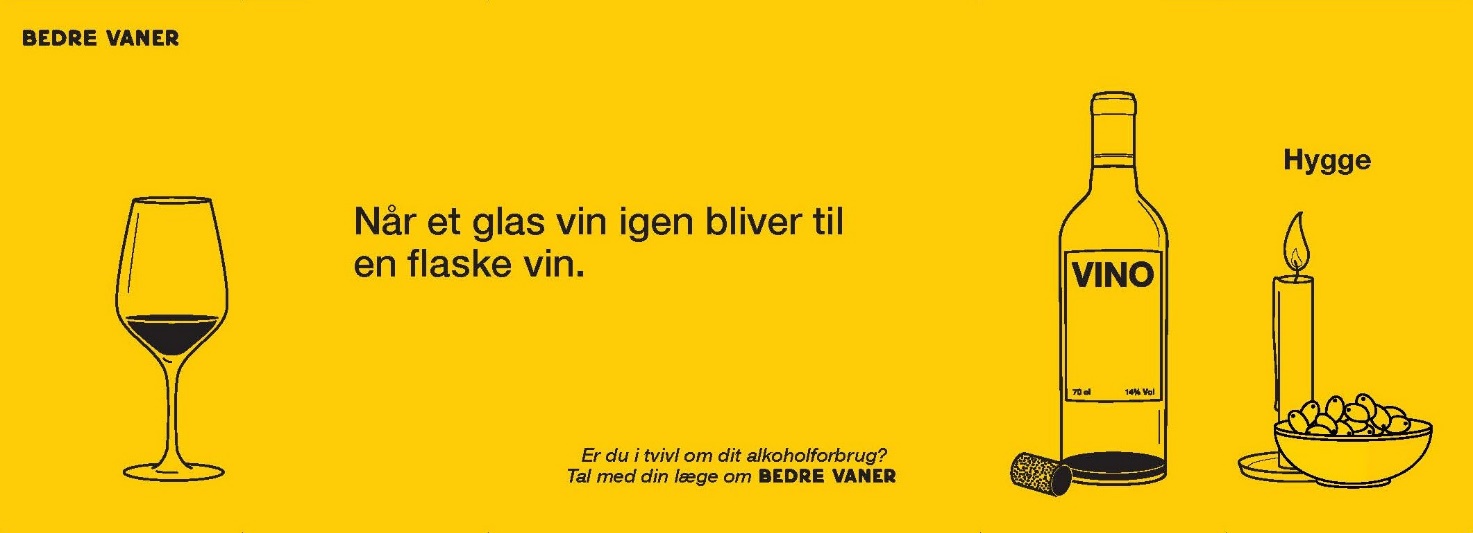


Front example 2.


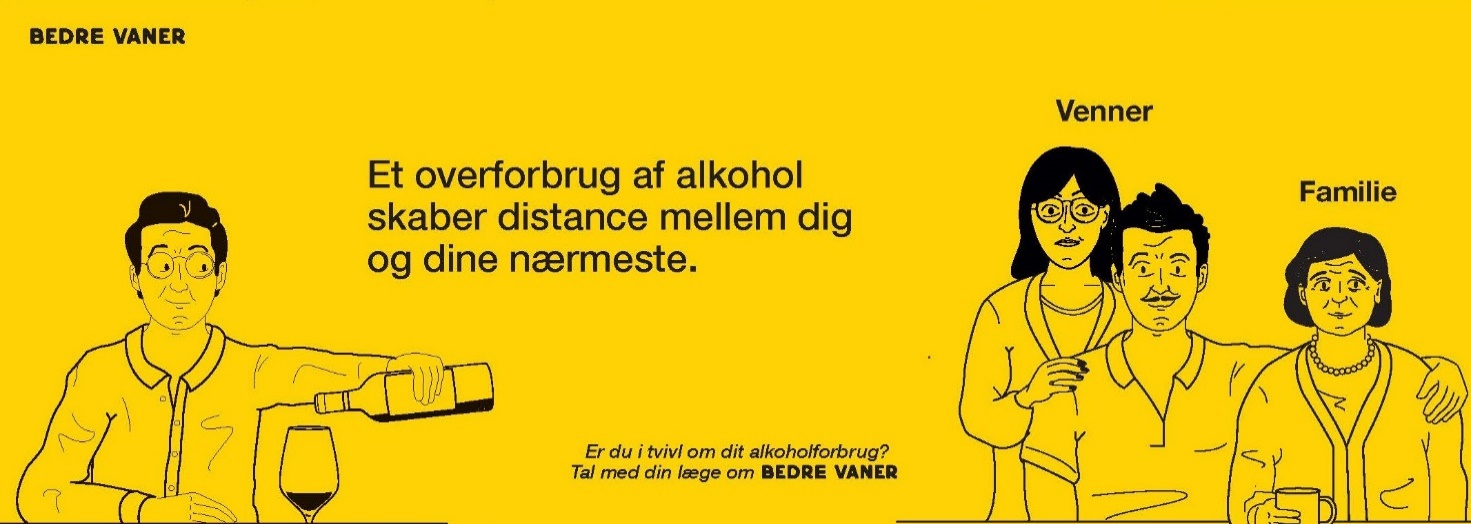


Back
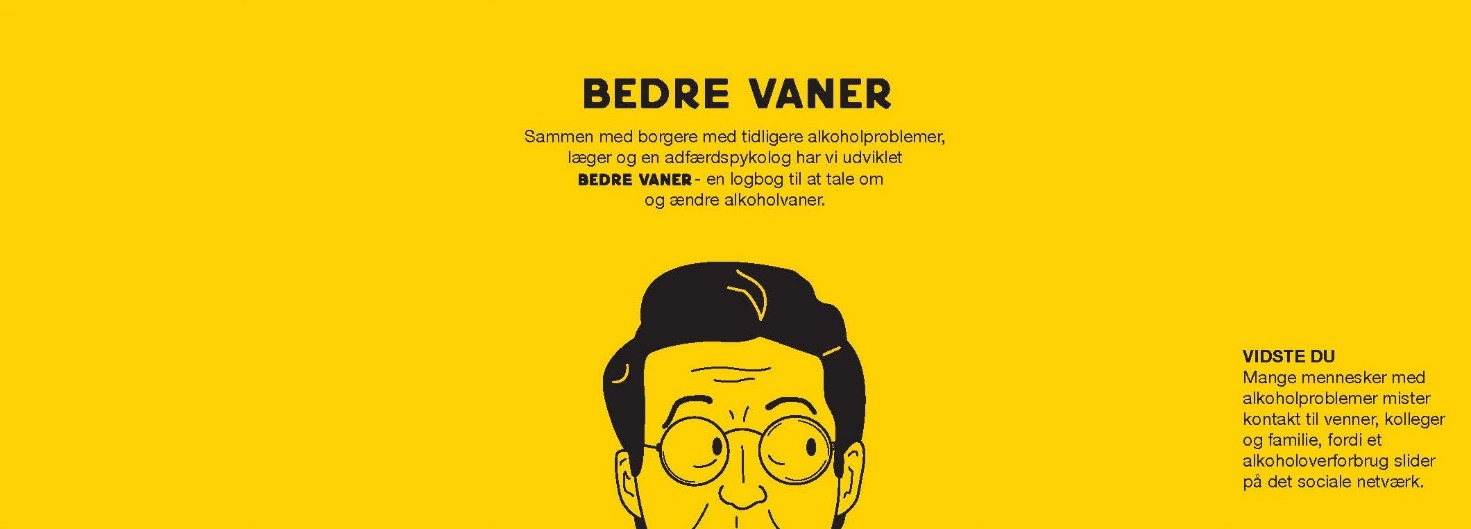


Translated text:

Front (1): “When a glass of wine again turns into a bottle of wine. Are you in doubt when it comes to your alcohol consumption? Talk to your doctor about BETTER HABITS” (“hygge” is a Danish word for coziness or having a good time).

Front (2): “An excessive use of alcohol creates distance between you and those closest to you.” (Subtext identical to nr. 1. Venner = friends, Familie = family)

Back: “BETTER HABITS. We created the BETTER HABITS logbook with input from citizens with prior alcohol problems, doctors, nurses, and a behavioral psychologist. It aims to help you improve your alcohol habits. If you’re interested, talk to your nurse or doctor”. Back, right side: brief information relevant to the “story” on the front of the flyers. In this case (flyer example 2): “Did you know: Many people with alcohol problems loose touch with their friends, colleagues, and family, because of the strain an excessive alcohol use can cause on one’s social network.”

The flyers fold in four places to create a fold-out story (see picture below) with a “surprise” element to the story. Example of bottles and flyers in a practice conference room:


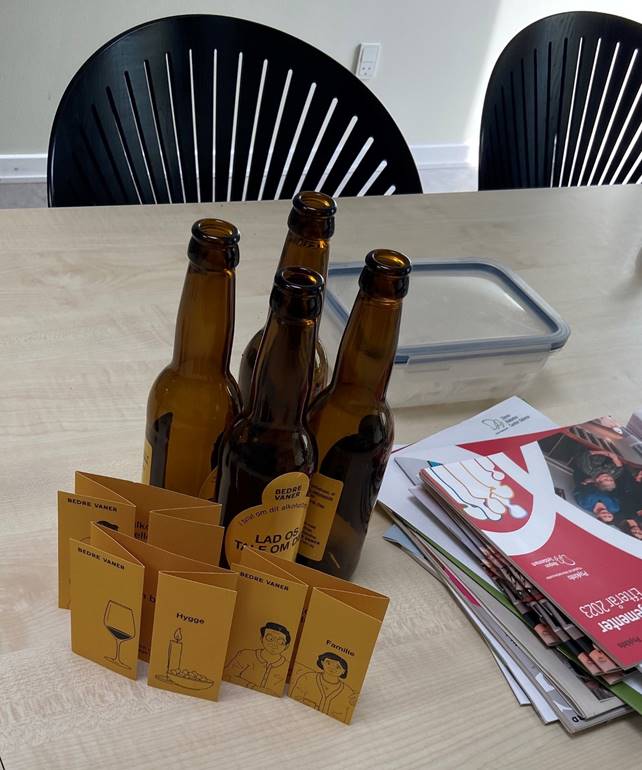


Bottles contained the following text:

(Front) “**Let’s talk about it**. Have you ever considered your alcohol intake? Read more on the back.”

(Back) “Did you know: The Danish Health Authorities recommend a maximum of 10 drinks per week and maximum of 4 drinks in one day. We have developed the logbook BETTER HABITS together with citizens with prior alcohol problems, doctors, nurses, and a behavioral psychologist. A logbook for talking about and changing alcohol habits”
