# Supplementary material for: Determinants of implementing the 15-method in Danish general practice using the consolidated framework for implementation science
Source: Addict Sci Clin Pract. 2025 May 16;20:43. doi: 10.1186/s13722-025-00571-0 (PMC12083036; doi:10.1186/s13722-025-00571-0)
Supplement: Supplementary file 2 — Supplementary Material 2 [file 13722_2025_571_MOESM2_ESM.docx]

Some questions are included as probes and follow-up questions and questions are omitted if answers are evident prior to the interview (e.g., information on title and role) Not all questions were presented in every interview. Lead (prioritized) questions are highlighted in bold and any role-specific questions are highlighted with the specified role in parentheses. After a lead question, the remaining questions under the same sub-heading can be used as probes or follow-up questions as needed.

# Introduction

*This interview aims to evaluate the implementation of the 15-method. We will discuss how it is used, whether it is used, and if you have changed the way you use it. We are also interested in ideas for improvements. The interviews will be recorded and transcribed. All information will be anonymized. (Oral consent is obtained at the beginning of interview).*

# Questions

1. **Background Information**

First, a few questions to help me understand who is involved in the project and the implementation of 15-method in this clinic.

- What is your title and role in the clinic?
- How are you involved in the iTAPP project? (user of the 15-method, coordinator, supervisor, super-user)
- How was it decided that you would participate in the project?
- **How was it decided that the method would be implemented in this clinic?**

**2. Open description of the implementation**
(An overview of the implementation process with a structural timeline to pinpoint milestones and to create a mental "event history calendar," which reduces recall bias and promotes sequential and parallel top-down memory recall (Belli, 1998))

- **I would like to hear about the process of implementing the 15-method in this clinic — in as much detail and with as many time points as possible: What are your thoughts and experiences with the method?**
- Has anything changed during the project?
- **Who in the clinic uses the method?**
- Who handles homework/reviews materials with patients?

**3. Domain-specific questions**

Now I would like to learn more about why you are participating and your general impression of the 15-method:

**(Innovation relative advantage Domain I)**

- **What do you think about using the method?**
- Does the method make it easier to ask about alcohol compared to what you used to do? If yes, how?
- To what extent does this intervention meet the actual need for an intervention in this area?

**(Tension for change Domain III)**

- **Has the method changed anything about how you discuss alcohol?** If something was particularly challenging before, has it become easier?

**(Innovation Evidence-base Domain I)**

- The 15-method has been tested abroad (Sweden), and we are testing its effectiveness here in Denmark. How significant is this for you?

**(Innovation Evidence-base Domain I; Innovation Design Domain I)**

- What do you think of the 15-method overall?
- What do you think about its credibility?

**(Complexity Domain I)**

- How does it work?
- **Is it easy or complex to use?**
- **What do you think about the structure of the method (the steps)?**
- Do you have the necessary overview?
- How does the material align with the idea/concept behind the method? (a stepwise flexible approach)

**(Innovation Design Domain I)**

- What do you think is good/less good about the quality, design, and layout of:
  - The manual?
  - The overview card?
  - The logbook?
  - The flyers?
  - The AUDIT form?

**(Relative Priority Domain III and Available Resources Domain III)**

- Do you have any other new/large initiatives in the clinic that are being implemented alongside this intervention?
- **How does this intervention/the use of the method rank compared to other activities in the clinic — is there time and capacity to use it?**
- Does the 15-method take time away from other tasks?
- Are these other projects/priorities short-term or long-term?

Now I would like to hear more about your experiences with implementing the 15-method in this clinic.
There are no right or wrong answers, and there is no single way to do it. I would also like to hear more about any changes you may have made or considerations/desires for changes you may have.

**(Access to Knowledge and Information Domain III)**

- Do you have access to the material/information you need to use the method?
- What is missing, if anything?
- **Have you received sufficient information and training in the method to start using it?**
- Is there a need for case examples and training before it is put into use?
- Is there sufficient communication with the project team for support?

**(Planning Domain V)**

- **How did you plan to implement the method in your daily work?**
- Can you describe the plan?
- Who did what?
- If there is a plan, do you have any milestones?
- How are you progressing toward these goals?

**(Compatibility Domain III)**

- **How does the method fit into your normal workflows?**
- Does it make any processes or types of work easier/harder and why?
- Which professional groups in the clinic use the method, and how does it work?
- How do you collaborate on this? (Teaming Domain V)
- What is the role of doctors compared to nurses? (Leadership engagement Domain III)
- If only participating nurses: Are the doctors aware of the project, and are there disadvantages/advantages in this? (nurse)
- Was anyone specifically designated, or did people volunteer?
- Has there been a shift in who uses the method the most compared to when you started?

**(Communications Domain III)**

- **How do you communicate about the project and the method — formally (meetings, emails) or informally (in the hallway, during breaks)?**
- What has been the most useful?
- Would you like more of the other (formal/informal)?
- Can you give examples related to the 15-method?

**(Work Infrastructure Domain III)**

- How do you divide the tasks related to the 15-method among you?
- Have you specified roles/responsibilities?

**(Roles Domain IV sub-domain)**

- **Is there support from the leadership for the other staff?**
- Is there one (or more) who leads the implementation of the method?
- Is there anyone who supports/helps others to implement the method, or is the work equally divided?
- Is there anyone in the clinic who is particularly good at managing projects or making things happen, and are they helping here?
- Who specifically in the clinic uses the 15-method? (deliverers, team members)

**(Adaptability Domain III)**

- **How do you adapt the method – if so, how?**
- Is the method flexible enough to fit into your daily routine? Why/why not?
- How could it better account for unique differences in the clinic?
- Are you actively doing something to adapt it?
- Have you made any physical or other changes to your environment to use the method (better)?

**(Complexity Domain I + Doing Domain V)**

- On a scale from 0 to 10, where 0 is the easiest thing in the world and 10 is impossible, how difficult would you say it was/is to implement the 15-method in your clinic?
- What made/makes it difficult?
- What makes it easier? What would make it even easier?
- Were there specific bottlenecks or significant points (made it harder/easier)?
- Have there been any unforeseen demands for time or other challenges that have consumed more resources than expected?

**(Doing Domain V)**

- If the method has been implemented:
- From your own perspective, on a scale from 0 to 10, where 0 is a failure and 10 is a success, how successful do you think the 15-method is in this clinic after it has been put into use?
- What do you base this assessment on?
  *NOTE: Focus here is on what participants perceive as success.*
- **What has been good/less good in terms of getting the method used in the clinic?**
- Have there been any major incidents or unforeseen changes/limitations that have affected the implementation or use? (critical incidents, Domain II)
- Are there any local factors that make it easier/harder to use the method (other initiatives in the municipality, collaboration with local institutions, socio-cultural aspects)?

**(Reflecting and evaluation Domain V)**

- **How (if at all) do you follow up on the implementation of the method in the clinic?**
- Have you brought it up in monthly/weekly meetings?
- Have you allocated time specifically for reflection or evaluation?

**(Assessing Needs Domain V)**
**(Doing Domain V; available resources Domain III)**

- What is necessary to ensure that a new method continues to be used (sustained use)?
  ... In general practice?
- **Do you feel that the necessary resources have been accounted for in this project?**

**(Innovation Cost Domain I)**

- What role does economics play?

**(Partnership and Connections Domain II)**

- **How do you assess your collaboration with organizations/institutions outside the clinic in this area? (referrals, municipal services)**

**(Capability Domain IV (sub-domain Characteristics), COM-B system)**

- **Do you feel that you have the skills and knowledge to use the method?**
- ... Skills to use the method the way you would like to?

**(Opportunity Domain V (sub-domain characteristics), COM-B system)**

- Do you feel that you have opportunities to change things in the clinic if you set your mind to it?
  For example, activities to strengthen skills (preferably related to the 15-method)?
- Opportunities to change conditions related to the 15-method (structure, workflows, material use)?

**(Motivation Domain V (sub-domain characteristics), COM-B system)**

- How important is the issue of alcohol in your practice?
- How ready are you to work on this issue?
- **What drives your engagement in working with the method?**
- Do you feel motivated in this area (alcohol)?

Now I will shift focus to the recipients/patients. I would like to hear your experiences/thoughts regarding patients' experiences.

**(Innovation Recipients Domain IV (sub-domain Roles); Need Domain IV (sub-domain Characteristics); Recipient Centeredness Domain III culture)**

- **What do you think the patients think about the method?**
- Do you have examples of specific experiences, conversations, or situations with patients?
- Do you have examples of specific expectations from the patients regarding the method?
- Do patients find it difficult/easy to follow the material/progression of the method?
- Have you experienced patients refusing to use the tools in the method? If so, do you know what led them to refuse?
- Are there elements that appeal to certain patients/situations?
- Are there parts that work particularly well for certain patients/consultations?

**Finally, considerations and suggestions for improving the method:**

- Do you have any ideas or considerations for improving the 15-method itself?
  The material (Manual, Logbook, Flyers, Overview card, AUDIT form)?
  The structure (steps)?
  Tips and tricks section?
  Supporting quotes?
  Digital options?
- Do you have any reflections on the introduction, training, and getting the method started in the clinic?
  Support, feedback?
  Assistance in getting the method incorporated into daily work/implementation support?
- Anything else you would like to comment on or have thoughts about?

Thank you

(closing remarks)
