# Supplementary material for: Determinants of implementing the 15-method in Danish general practice using the consolidated framework for implementation science
Source: Addict Sci Clin Pract. 2025 May 16;20:43. doi: 10.1186/s13722-025-00571-0 (PMC12083036; doi:10.1186/s13722-025-00571-0)
Supplement: Supplementary file 3 — Supplementary Material 3 [file 13722_2025_571_MOESM3_ESM.docx]

Recommended strategies based on the Expert Recommendations for Implementing Change (ERIC) project (1, 2) using the Consolidated Framework for Implementation Research (CFIR)-ERIC Implementation Strategy Matching Tool provided at [www.cfirguide.org](http://www.cfirguide.org).

The CFIR-ERIC matching tool was originally mapped on to the 2009 version of CFIR, which has since been updated (3). Not all of the updated ERIC strategies map onto the updated CFIR determinants (4) (<https://cfirguide.org/choosing-strategies/>).

The ERIC matching tool supplies “level 1” and “level 2” strategies. A level 1 strategy is a strategy endorsed by more than half of the experts (N=25) who participated in the study by Waltz et al. 2019 (4). Variables influencing the ranking include the strategy’s relevance, improvement opportunity (will it make an impact), feasibility, validity (evidence base), and level of difficulty. Below, we present the top three recommended strategies to address identified barriers and mixed determinants for implementing the 15-method in Danish general practice using the CFIR-ERIC Implementation Strategy Matching Tool.
Level 1 strategies are highlighted with *.

*Note: “Recommend strategies” should be considered preliminary suggestions for strategies in relation to the present study. Robust implementation outcome measures are needed before specific strategies can be proposed.*

| **Identified barriers for implementing the 15-method** | **Top three recommended strategies to address the identified determinant** |
| --- | --- |
| Local Attitudes | Determinant not mapped onto ERIC implementation strategies |
| Local Conditions | Determinant not mapped onto ERIC implementation strategies |
| Partnerships & Connections | 1. Build a coalition *  2. Develop academic partnerships *  3. Promote network weaving * |
| Structural Characteristics - Work Infrastructure | 1. Assess for readiness and identify barriers and facilitators  2. Build a coalition  3. Identify and prepare champions |
| Planning | 1. Develop a formal implementation blueprint *  2. Conduct local needs assessment *  3. Assess for readiness and identify barriers and facilitators |
| **Determinants with mixed influence on implementing the 15-method** |  |
| Innovation Complexity | 1. Develop a formal implementation blueprint  2. Promote adaptability  3. Conduct cyclical small tests of change / Conduct ongoing training (shared 3^rd^) |
| Innovation Design | 1. Promote adaptability  2. Develop educational materials  3. Obtain and use patients/consumers and family feedback / Develop and implement tools for quality monitoring (shared 3^rd^) |
| Innovation Cost | 1. Access new funding *  2. Alter incentive/allowance structures  3. Develop resource sharing agreements /  Make billing easier (shared 3^rd^) |
| Relative Priority | 1. Conduct local consensus discussions  2. Alter incentive/allowance structures  3. Assess for readiness and identify barriers and facilitators |
| Incentive Systems | 1. Alter incentive/allowance structures *  2. Access new funding  3. Identify and prepare champions / Use other payment schemes (shared 3^rd^) |
| Assessing Needs – Innovation Deliverers | Determinant not mapped onto ERIC implementation strategies |
| Engaging – Deliverers | 1. Identify and prepare champions *  2. Conduct local consensus discussions  3. Assess for readiness and identify barriers and facilitators |
| Engaging – Recipients | 1. Involve patients/consumers and family members *  2. Prepare patients/consumers to be active participants *  3. Intervene with patients/consumers to enhance uptake & adherence * |
| Doing | 1. Purposely reexamine the implementation  2. Assess for readiness and identify barriers and facilitators / Develop and implement tools for quality monitoring / Provide local technical assistance (shared 2^nd^)  3. Develop a formal implementation blueprint /  Conduct ongoing training (shared 3^rd^) |
| Reflecting and Evaluating – Implementation | 1. Develop and implement tools for quality monitoring *  2. Audit and provide feedback *  3. Develop and organize quality monitoring systems |

An elaboration on the presented preliminary strategies including ancillary material is presented in Powell, Waltz et al. 2015 additional file 6 (1).

# Literature

1. Powell BJ, Waltz TJ, Chinman MJ, Damschroder LJ, Smith JL, Matthieu MM, et al. A refined compilation of implementation strategies: results from the Expert Recommendations for Implementing Change (ERIC) project. Implementation Science. 2015;10(1):1-14.

2. Waltz TJ, Powell BJ, Matthieu MM, Damschroder LJ, Chinman MJ, Smith JL, et al. Use of concept mapping to characterize relationships among implementation strategies and assess their feasibility and importance: results from the Expert Recommendations for Implementing Change (ERIC) study. Implement Sci. 2015;10:109.

3. Damschroder LJ, Reardon CM, Widerquist MAO, Lowery J. The updated Consolidated Framework for Implementation Research based on user feedback. Implementation Science. 2022;17(1).

4. Waltz TJ, Powell BJ, Fernández ME, Abadie B, Damschroder LJ. Choosing implementation strategies to address contextual barriers: diversity in recommendations and future directions. Implement Sci. 2019;14(1):42.
